# Relationships between the expectations based on the regularity of preceding sound sequences and the medial olivocochlear reflex

**DOI:** 10.1371/journal.pone.0304027

**Published:** 2024-07-17

**Authors:** Yuki Ishizaka, Sho Otsuka, Seiji Nakagawa

**Affiliations:** 1 Department of Medical Engineering, Graduate School of Science and Engineering, Chiba University, Chiba, Japan; 2 Center for Frontier Medical Engineering, Chiba University, Chiba, Japan; 3 Med-Tech Link Center, Chiba University Hospital, Chiba, Japan; Universidad de Chile, CHILE

## Abstract

Rhythms are the most natural cue for temporal anticipation because many sounds in our living environment have rhythmic structures. Humans have cortical mechanisms that can predict the arrival of the next sound based on rhythm and periodicity. Herein, we showed that temporal anticipation, based on the regularity of sound sequences, modulates peripheral auditory responses via efferent innervation. The medial olivocochlear reflex (MOCR), a sound-activated efferent feedback mechanism that controls outer hair cell motility, was inferred noninvasively by measuring the suppression of otoacoustic emissions (OAE). First, OAE suppression was compared between conditions in which sound sequences preceding the MOCR elicitor were presented at regular (predictable condition) or irregular (unpredictable condition) intervals. We found that OAE suppression in the predictable condition was stronger than that in the unpredictable condition. This implies that the MOCR is strengthened by the regularity of preceding sound sequences. In addition, to examine how many regularly presented preceding sounds are required to enhance the MOCR, we compared OAE suppression within stimulus sequences with 0–3 preceding tones. The OAE suppression was strengthened only when there were at least three regular preceding tones. This suggests that the MOCR was not automatically enhanced by a single stimulus presented immediately before the MOCR elicitor, but rather that it was enhanced by the regularity of the preceding sound sequences.

## Introduction

Most sounds in our daily surroundings (i.e., music, waves, and construction drills) have rhythmic structures; thus, rhythms are the most natural cues for predicting the timing of future sound occurrences. Previous studies have examined how rhythms modulate perceptual auditory excitability over time [1, 2]. Jones et al. demonstrated better discriminatory performance for auditory targets coinciding with the predicted beat of the rhythm, with performance falling off systematically as the target occurs increasingly early or late [1].

Rhythm-induced expectations have been shown to have behavioral advantages even when they do not notify the timing of the target, suggesting that temporal expectation driven by regular rhythms may operate through an automatic, exogenous process [3]. This notion was corroborated by findings that showed that reaction time is not influenced by the addition of an interference task when predicting targets from a rhythmic stream [4]. The automatic nature of the tracking process dissociated the rhythmic temporal expectation from the endogenous temporal orientation observed in a cue task, in which a target stimulus appeared after a symbolic cue that allowed listeners to predict the timing of the target appearance. Temporal cues produce significant advantages in behavioral task outcomes compared to neutral cues [5], and cue-based temporal orienting demands controlled attention [6].

Helfrich et al. reported that the functional structure of top-down attention is rhythmic [7]. Moreover, studies reported that slow-rate cortical oscillations synchronize to regularly presented sound stimuli, increasing the response to arriving sounds [8] and phase synchronization of cortical neural activity at the stream frequency [9–11]. Further, a recent study showed that in the human brainstem, the response to acoustic stimuli presented at regular intervals was stronger than that presented at random intervals [12]. However, the stages modulated by the cortical rhythmic temporal expectation remain unclear.

Regarding endogenous temporal orientation, Otsuka et al. recently reported that auditory peripheral activity is modulated via the medial olivocochlear reflex (MOCR) by the presentation of visual stimuli that convey the timing of stimulus sound appearances [13]. Medial olivocochlear (MOC) fibers are efferent projections from the superior olivary complex to the outer hair cells (OHCs), which are activated by acoustic stimulation and exert an inhibitory effect on OHCs [14, 15]; this effect has been termed the MOCR. The MOCR improves signal detection in noisy environments by preventing auditory nerve adaptation to the noise [16, 17]. Additionally, the MOCR is thought to play an important role in protecting the auditory system from acoustic overexposure [18, 19].

Interestingly, efferent fibers from cortical regions project to the MOC bundle via subcortical nuclei [15, 20, 21] and mediate top-down control of the MOCR [22]. The MOCR may be affected by endogenous orientation to visual [23, 24] and auditory [25] targets, and specific laterality [26]. Furthermore, an interaction of low-frequency cortical oscillations with auditory peripheral responses (otoacoustic emissions) during tasks requiring visual and auditory selective attention has also been reported [27], supporting the hypothesis of cortical modulation of peripheral activities via corticofugal pathways. On the other hand, there are some studies that are negative to the existence of MOC-induced inhibition by attention. Francis et al. (2018) reported that noise levels in the ear canal decrease when performing an auditory attention task [28]. Bell and Jedrzejczak (2021) also reported that the reduction of auditory peripheral response measured in the ear canal during a selective attention task may be attributed to the reflex contraction of muscles in and near the ear [29].

Riecke et al. [30] recently reported that auditory peripheral responses increased when the frequency of a behaviorally relevant upcoming sound is predictable from monotonic (ascending or descending) frequency changes in isochronous tone sequences. However, no studies have examined whether changes in MOCR associated with rhythmic, presumably automatic, and exogenous, temporal expectation can modulate peripheral auditory processing.

Thus, to determine whether the MOCR is modulated by anticipating the arrival of the MOCR elicitor based on the regularity of sound sequences preceding the MOCR elicitor, we first compared the strength of the MOCR between conditions in which the preceding sounds were presented with a fixed and variable inter-stimulus interval (ISI) (Experiment 1). Second, we investigated the number of preceding sounds required to trigger the effects of rhythmic temporal expectations on the MOCR (Experiment 2).

## Method

### Participants

Thirteen volunteers (nine men and four women), aged 18–25 years (mean = 22.2, standard deviation = 1.80) participated in Experiment 1, and 11 volunteers (five men and six women), aged 18–25 years (mean = 21.9, standard deviation = 1.78) in Experiment 2. All ears had normal pure-tone audiometric thresholds (HL< 20 dB) from 0.25 to 8 kHz. The experiments were approved by the Institutional Review Board of Life Science Research of Chiba University. All participants provided written informed consent before the experiments. We recruited the participants between May 2019 to August 2021 and had access to information that could identify individual participants during or after data collection.

### Equipment

Stimuli were digitally synthesized at a sampling rate of 48 kHz and converted to analog signals using a Roland OCTA-CAPTURE system (16 bits). The analog signals were amplified by a headphone buffer and presented through Etymotic Research ER-2 earphones connected to an ER-10B+ low-noise microphone system. Prior to the measurements, outputs from the ER-2 were calibrated using a DB2012 accessory (external ear simulator) of a Bruel and Kjaer Type 4257 ear simulator (IEC 711). The ear canal sound pressure was recorded with an Etymotic Research ER-10B+ low-noise microphone system inserted in each ear. A Roland OCTA-CAPTURE system was used for the analog-to-digital conversion (48 kHz, 16 bits).

### Evaluation of MOCR

We evaluated the MOCR noninvasively using otoacoustic emission (OAE), which are energy byproducts generated by the OHC motility that leak back out into the ear canal and measured as sound [31]. Since the MOC projects to the OHCs in both ears, the OHC amplification in the contralateral ear are inhibited when noise is presented to one ear [32]. We measured the suppression of click-evoked otoacoustic emissions (CEOAEs) induced by noise presented to the contralateral ear, referred to as the MOCR elicitor. The amount of suppression was assumed to be related to the MOCR strength [32].

**Fig 1:**
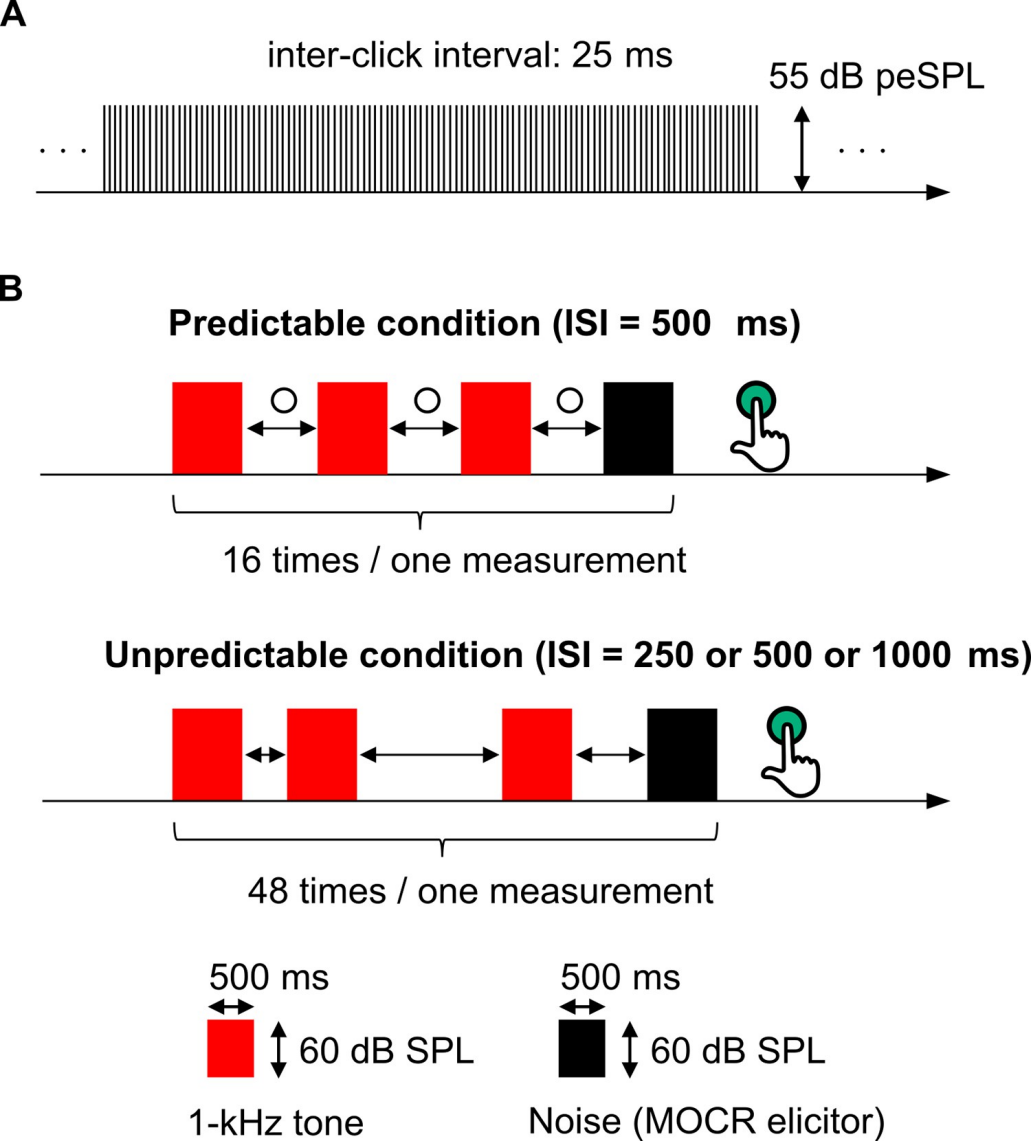
Schematic illustration of the sound sequences in Experiment 1. (A) Clicks to evoke the otoacoustic emissions presented to the right ear. (B) Stimulus sound sequence composed of one noise, referred to the medial olivocochlear reflex (MOCR) elicitor, and three tone bursts presented to the left ear. The tone bursts preceding the MOCR elicitor were presented at regular (predictable condition) or irregular interval (unpredictable condition) Participants were instructed to press the button once after the presentation of the MOCR elicitor to ensure sustained attention to the presented sound.

In the right ear, clicks were presented at a 55-dB peak-equivalent sound pressure level (SPL). The inter-click interval of the clicks was 25 ms and each click had a duration of 100 μs (Fig 1A). The left ear was presented with the MOCR elicitor (noise to elicit the MOCR) at 60 dB SPL. The noise was band-pass filtered between 100 and 10,000 Hz, with a duration of 0.5 s, including a 10-ms raised-cosine ramp. It has been reported that weak MOCRs are elicited when attention is directed to the ear presented with the OAE-evoked stimulus [33, 34]. In this experiment, it was necessary to focus attention on the regularity of the stimulus sound sequence presented to the left ear and not on the clicks presented to the right ear. Intermittent presentation of click trains may capture the participants’ attention, therefore, the clicks were presented continuously during the measurement. The onset of the MOCR elicitor was synchronized with a click presentation; thus, clicks were presented 20 times during the MOCR elicitor presentation. In experiments 1 and 2, we presented 0–3 tone bursts regularly or irregularly prior to the MOCR elicitor and examined the effects of the regularity of the preceding sound sequences on the MOCR.

### Experiment 1

We compared MOCR strengths between conditions in which the sound sequence preceding the MOCR elicitor was regular or irregular. One stimulus block presented to the left ear comprised a MOCR elicitor and three tone bursts that preceded the MOCR elicitor. The tone burst had a frequency of 1 kHz with a duration of 0.5 s, including a 10-ms raised-cosine ramp. The ISI between the tone bursts and the MOCR elicitor varied in two conditions: Predictable and Unpredictable (Fig 1B). In the Predictable condition, the ISIs between the three tone bursts and the MOCR elicitor were set to 500 ms. In the Unpredictable condition, the tone bursts and MOCR elicitor were presented with a variable ISI randomly chosen from 250, 500, and 1000 ms for each presentation, and the average ISI of all stimulus blocks was 500 ms. The number of blocks for the Predictable and Unpredictable conditions presented during one measurement session was 16 and 48, respectively. The blocks during the Predictable and Unpredictable conditions were presented randomly. In the Unpredictable condition, the number of blocks in which the ISI of the third tone burst and the MOCR elicitor was 250, 500, and 1000 ms were all 16. The intertrial interval of the blocks randomly varied from 3.0 to 6.0 s. The measurement session was repeated four times and the total duration of the OAE measurements for each participant was approximately 60 min, including a 10-min break after each measurement session.

We compared the suppression of OAEs induced by the MOCR elicitor between the Unpredictable and Predictable conditions. The comparison of OAE suppression was performed for the responses recorded in the blocks with the same ISI between the third tone burst and the MOCR elicitor, that is, 500 ms. Hence, we obtained responses in 16 blocks for the comparison per one measurement session both in the Predictable and Unpredictable conditions. As it is necessary for listeners to listen to each stimulus blocks at least once for predicting the timing of the arrival of the MOCR elicitor, the first presented block in each measurement session was deleted from the analysis for each condition. Therefore, responses obtained in 60 blocks were pooled for each condition as a total of the four measurement sessions. Participants were instructed to press the button after every presentation of the MOCR elicitor to direct their attention to the sound sequences presented to the left ear.

### Experiment 2

To examine how many preceding sounds are needed to trigger the effects of rhythmic temporal expectations on the MOCR, we compared the MOCR strength among conditions with the different number of preceding sounds:

1. Non-preceding-sound condition: Only the MOCR elicitor was presented.
2. One-preceding-sound condition: An MOCR elicitor and the tone preceding it were presented.
3. Two-preceding-sounds condition: An MOCR elicitor and two tones preceding it were presented
4. Three-preceding-sounds condition: An MOCR elicitor and three tones preceding it were presented.

**Fig 2:**
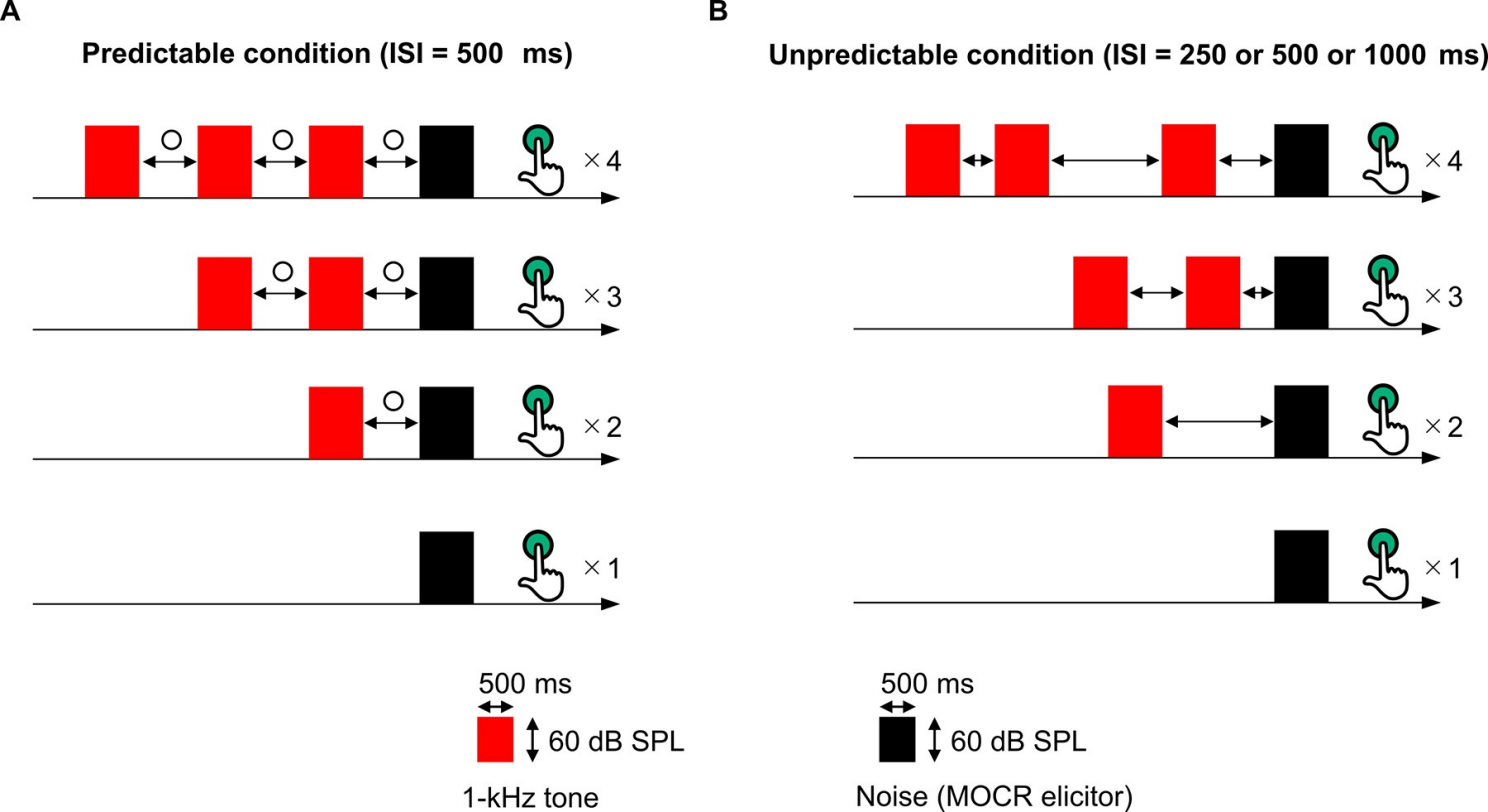
Schematic illustration of the sound sequences presented to the left ear in Experiment 2. The number of tone bursts preceding to an MOCR elicitor, was varied from zero (only an MOCR elicitor was presented) to three. The tone bursts were presented at regular interval in the Predictable condition (A) and at irregular interval in the Unpredictable condition (B). Participants were instructed to press the button after the presentation of the MOCR elicitor to ensure sustained attention to the presented sound, and the number of the button press was varied from one to four corresponding to the number of the preceding tone bursts.

The OAE measurements were performed separately in the Predictable condition in which the ISI was fixed at 500 ms (Fig 2A) and in the Unpredictable condition in which the ISI was randomly chosen from 250, 500, or 1000 ms (Fig 2B). In both the Predictable and Unpredictable conditions, each of the four types of stimuli blocks was presented 24 times per measurement session in a randomized order. The measurement session was performed thrice under Predictable and Unpredictable conditions, respectively. As responses obtained in the first presented block of the 24 blocks were removed in each measurement session, responses obtained in 69 blocks were pooled for each condition as a total of the three measurement sessions. The total duration of the OAE measurements for each participant was approximately 90 min, including a 10-min break after each measurement session. Participants were instructed to press the button after every presentation of the MOCR elicitor to direct their attention to the sound sequences.

### Recording and analysis

Recorded CEOAE waveforms obtained in each block were bandpass filtered between 1 and 3 kHz, where the largest MOCR-related CEOAE suppression was observed [35]. Epochs with a duration of 0.5 s pre- and post-onset of the MOCR elicitor, which included 20 CEOAE waveforms, respectively, were extracted from the filtered signals for each block. All the extracted CEOAE waveforms obtained in the pre- and post-onset epochs were averaged across blocks for each condition. The root-mean-square (RMS) of the 5–25 ms region of each averaged CEOAE waveform was calculated and is referred to as *P*^*pre−onset*^ and *P*^*post−onset*^, respectively (in Pa). The MOCR strength was defined using Eq (1), as shown below.


$$-20\;\log_{10}\frac{P^{post-onset}}{P^{pre-onset}}$$ (1)

It should be noted that OAEs originate in the cochlea and are treated as sounds propagating through the middle ear. Therefore, the measured OAEs can be influenced by the middle ear muscle reflexes (MEMR). However, previous studies have shown that, at noise levels up to 60 dB SPL, the MEMR does not contribute to the OAE suppression induced by contralateral acoustic stimulation in the normal ear [36]. Hence, it can be assumed that in our experiment, the OAE suppression and its changes is not influenced by the MEMR.

To ascertain this, we evaluated the MEMR simultaneously. The blocks used to evaluate MEMR strength were the same as the blocks used to calculate the MOCR strength. The initial portion (0–4 ms) of responses to the clicks in the pre-onset and post-onset epochs of the MOCR elicitor were extracted. The initial portions of the waveforms are dominated by the ringing of the click stimulus inside the ear canal. Therefore, their changes associated with the MOCR elicitor presentation can be assumed to reflect MEMR-related changes in the reflectance of the eardrum or middle ear transmission.

Firstly, we tested whether MEMR was evoked by the presentation of the MOCR elicitor, because the MEMR activation causes change in impedance characteristics of the middle ear system [37, 38]. Band-pass filters with 1/3-octave bandwidths were applied to the responses at the center frequency (*f*_*c*_) of 100, 125, 160, 200, 250, 315, 400, 500, 630, 800, 1000, 1250, 1600, 2000, 2500, 3150 and 4000 Hz. Thereafter, all the filtered waveforms in the pre- and post- conditions were averaged respectively across blocks for each condition and each center frequency. RMS for the initial portion of each averaged waveform was calculated and is referred to as ${\;}^{fc}P_{init}^{pre-onset}$ and ${\;}^{fc}P_{init}^{post-onset}$, respectively (in Pa). Finally, the ${\;}^{fc}P_{init}^{pre-onset}$ and ${\;}^{fc}P_{init}^{post-onset}$ were compared for each center frequency.

We also analyzed the correlation between MEMR and MOCR as described by Boothalingam et al. [39]. The MEMR at each frequency was defined as the level difference between the pre- and post-onset condition (^*fc*^Δ*L*_*init*_), which was calculated by substituting ${\;}^{fc}P_{init}^{pre-onset}$ and ${\;}^{fc}P_{init}^{post-onset}$ to Eq (1). To evaluate the MOCR at the corresponding frequencies, the 5–25 ms region of the responses, in which OAE was mainly observed, was extracted after applying a band-pass filter to the signal in 1/3-octave bandwidth (*f*_*c*_ = 1000, 1250, 1600, 2000, 2500, 3150, 4000 Hz, where significant MOCR effect is observed). RMS for the extracted waveform was calculated and is referred to as ^*fc*^*P*^*pre−onset*^ and ^*fc*^*P*^*post−onset*^, respectively (in Pa). The level difference between the pre- and post MOCR elicitor condition at each center frequency (^*fc*^Δ*L*_*OAE*_) was calculated for each subject from Eq (1). Pearson’s correlation coefficients between ^*fc*^Δ*L*_*OAE*_ and ^*fc*^Δ*L*_*init*_ were calculated for each center frequency.

The signal-to-noise ratio (SNR) was calculated to confirm that the highest quality OAEs were measured. First, the even-numbered click waveforms obtained in each post-onset epoch were reversed, and then averaged together with the odd-numbered click waveforms. This process cancels the CEOAEs recorded in the ear canal. This value corresponds to the sound pressure level of the noise floor (*P*_*noise floor*_). Finally, the SNR was calculated from Eq (1) by substituting *p*^*pre−onset*^ for *p*_*noise floor*_. As a result, the SNR ≥ 15 dB was obtained for all subjects in every condition of experiments 1 and 2.

### Statistical analysis

In experiment 1, a paired t-test was performed across the Predictable and Unpredictable conditions. In experiment 2, since measurements for the blocks of the Predictable and Unpredictable condition were conducted in separate measurement sensations, a one-factor repeated-measures analysis of variance (ANOVA) was performed with the number of preceding sounds as a factor.

To test whether significant MEMR was elicited, a two-factor repeated-measures ANOVA was performed with the center frequencies and the presentation of the MOCR elicitor as factors in both the Predictable and Unpredictable conditions in the experiment 1. A three-factor repeated-measures ANOVA was performed with the center frequencies, the presentation of the MOCR elicitor and the number of preceding sounds as factors in both the Predictable and Unpredictable conditions in the experiment 2. As for the associations between MEMR and MOCR effects, Pearson’s correlation coefficient (r) and the p-value were calculated. In all analyses, the criterion of significance was set to p < 0.05.

## Results

### Experiment 1

**Fig 3:**
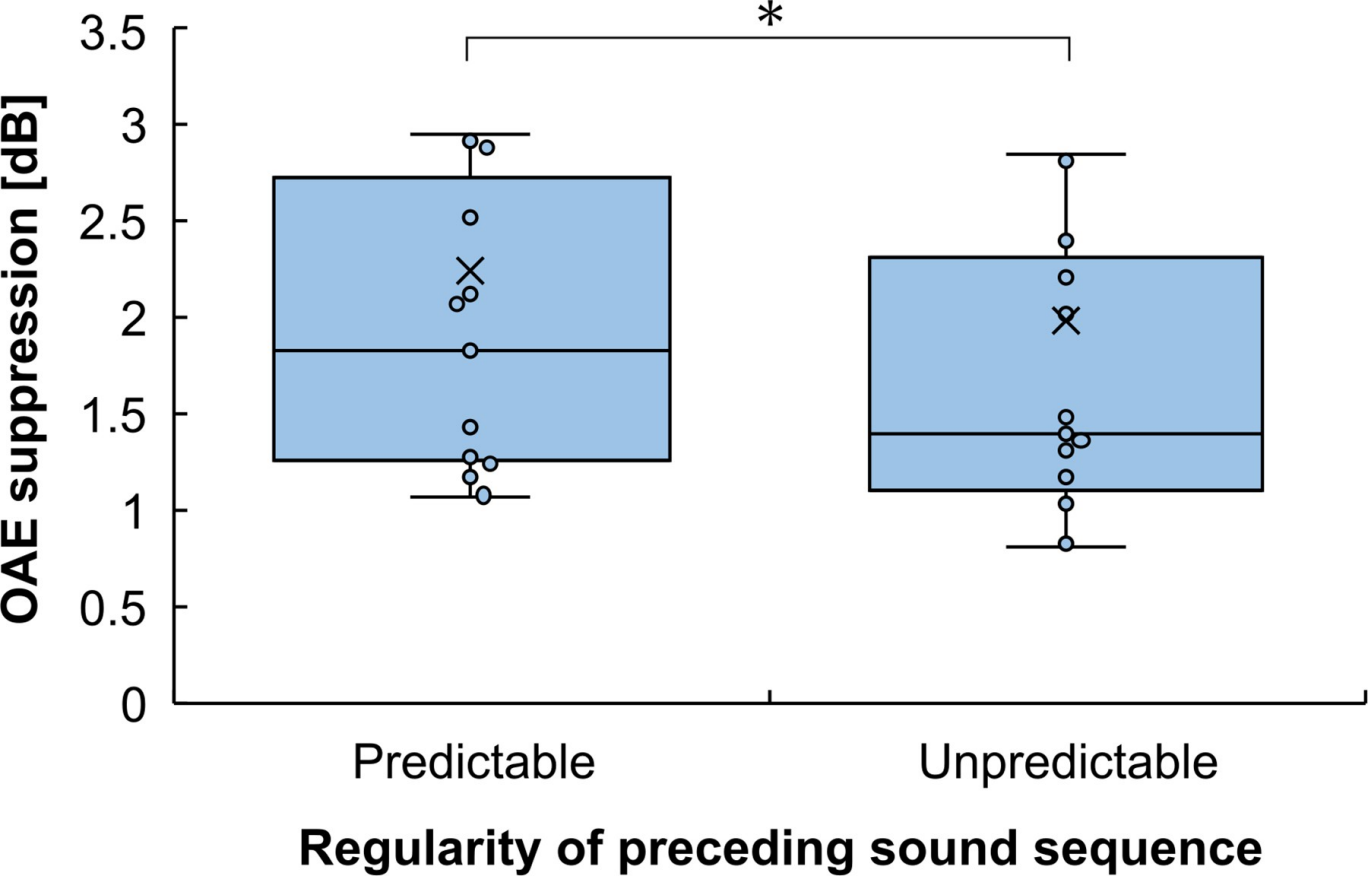
Mean otoacoustic emissions suppression in Experiment 1. The MOCR induced by the Predictable condition was stronger than that induced by the Unpredictable condition. Error bars represent the standard error of the mean. *p < 0.05 (paired t-test).

The extent of OAE suppression according to the presence of contralateral noise in each condition is shown in Fig 3. The average OAE suppressions were 1.88 dB (SD = 1.58) and 1.77 dB (SD = 1.63) for the Predictable and Unpredictable conditions, respectively. To compare the strengths of the MOCR induced by the MOCR elicitor between Predictable and Unpredictable conditions, a paired t-test was performed across the two conditions which showed a significant difference between the MOCR strengths in both conditions (t (12) = -2.31, p = 0.040). Each participant’s data is shown in S1 Table.

**Fig 4:**
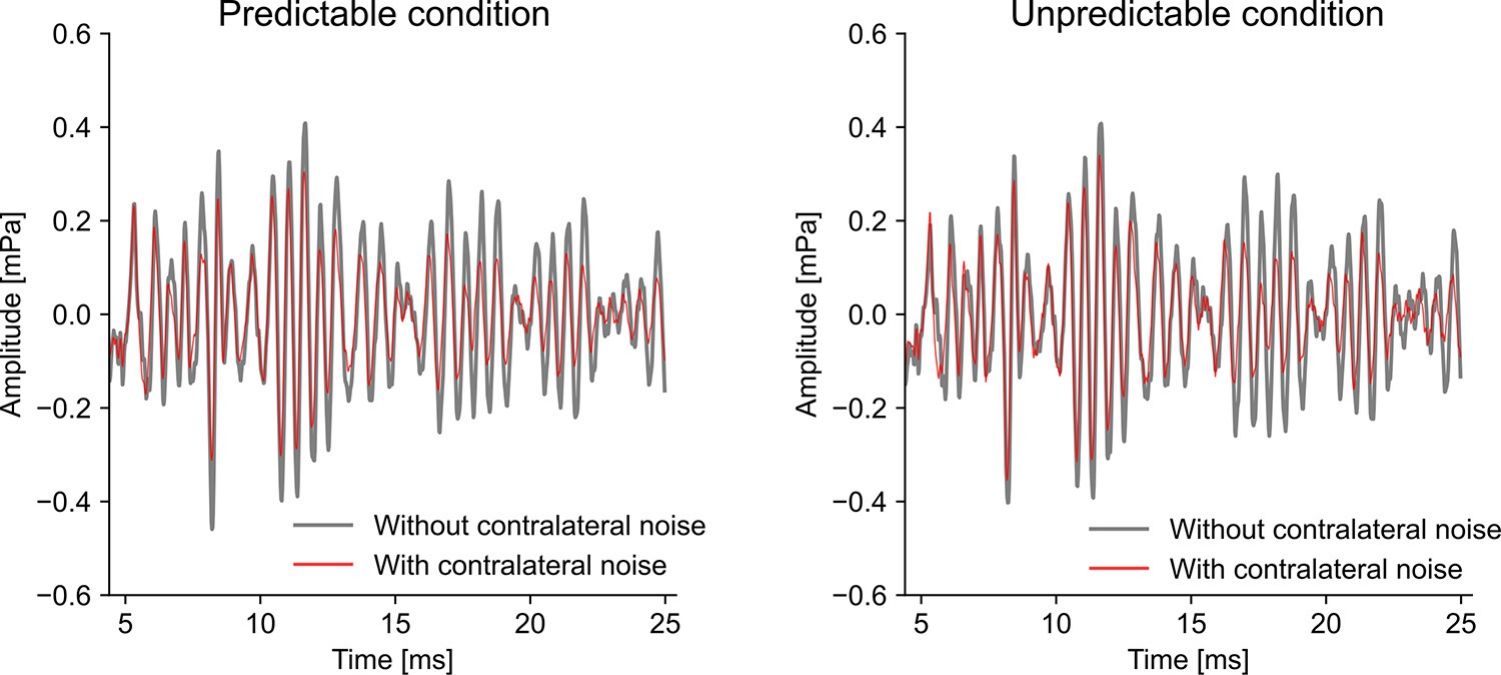
A typical example of the click evoked otoacoustic emission (CEOAE) in Predictable and Unpredictable condition. These CEOAE waveforms were taken from subject 14 in the experiment 1. The 5–25 ms interval in which OAE is mainly observed is shown. The gray and red lines show the OAE without and with contralateral noise conditions, respectively. Each response is the averaged result of 1200 clicks.

Fig 4 shows a typical example of the 5–25 ms region of the CEOAE waveforms in the Predictable and Unpredictable with and without contralateral MOCR elicitor. In both Predictable and Unpredictable conditions, the suppression of the OAE amplitude can be observed in the with contralateral noise conditions compared to the without contralateral noise conditions.

**Fig 5:**
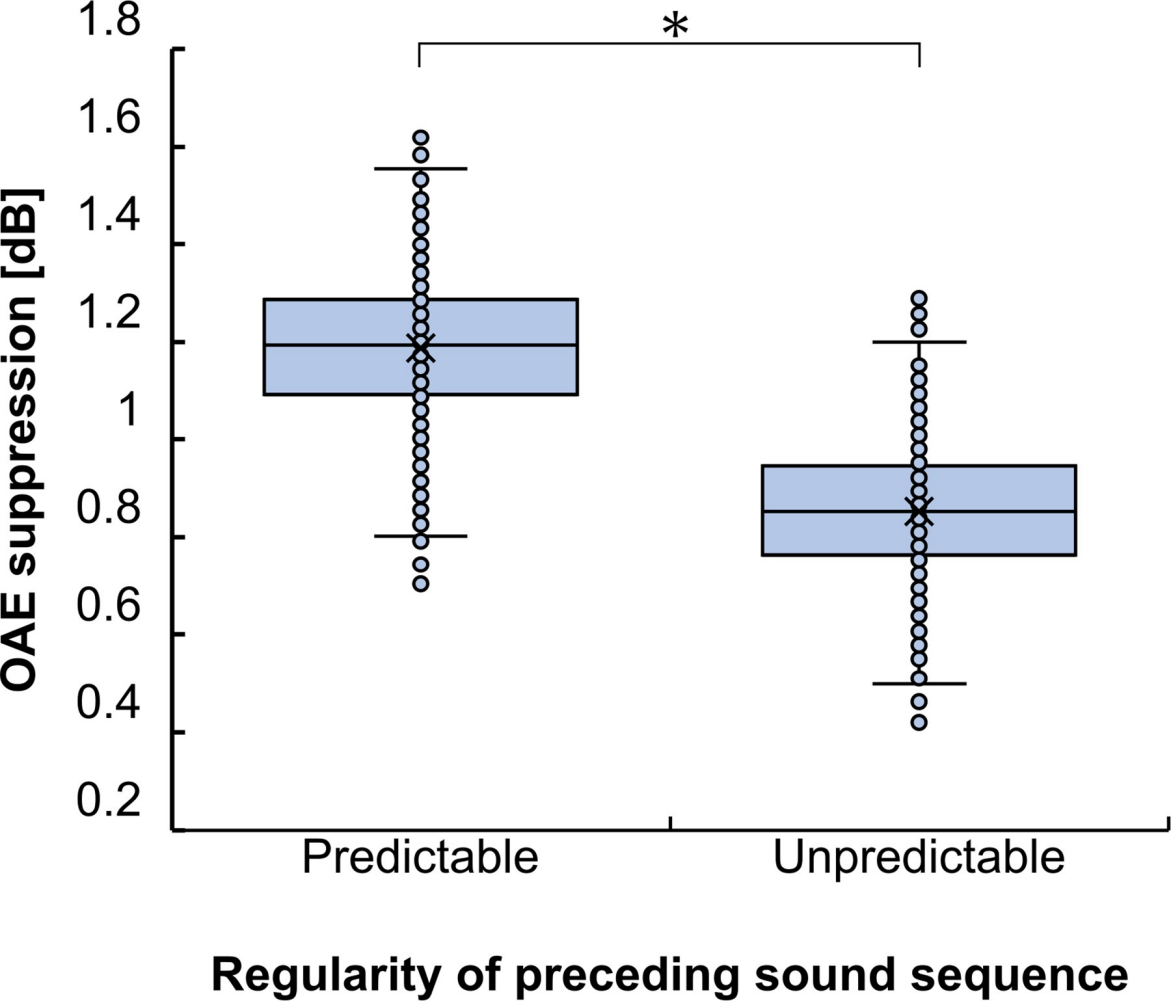
The distribution of the bootstrap means of otoacoustic emission (OAE) suppression in the Predictable and Unpredictable condition for subject 11. *p<0.05: the lower limit of the 95% confidence interval (CI) of the distribution of the difference between bootstrap mean of two conditions (Predictable minus Unpredictable) was positive.

To evaluate the significance in the difference of the OAE suppression between the Predictable and Unpredictable conditions within subjects, a bootstrap method was used. Specifically, we randomly extracted 1200 responses from 1200 responses with repetition and calculated the mean of them, referred to a bootstrap mean, for the Predictable and Unpredictable condition. This procedure was repeated 1000 time. The distribution of the bootstrap means in the Predictable and Unpredictable conditions for subject 11 was shown in Fig 5 as a typical example. Subsequently, the distribution of the difference between each bootstrap mean of two conditions (Predictable minus Unpredictable) was calculated. When the lower limit of the 95% confidence interval (CI) of the distribution was bigger than zero, it followed that the OAE suppression in the Predictable condition was significantly stronger than that in the Unpredictable condition. The estimated lower and upper limit of the 95% CI of the distributions for subject 11 was 0.046 and 0.65 dB, respectively. The positive lower limit of the 95% CI ensures that the OAE suppression in the Predictable condition was significantly stronger than that in the Unpredictable condition for subject 11. Similarly, seven of the thirteen participants showed significantly increased OAE suppression in the Predictable condition.

To test whether significant MEMR was induced by the MOCR elicitor, the sound pressure of initial portion of the OAE waveforms between with and without contralateral MOCR elicitor was compared. A repeated-measures ANOVA with the center frequency as a factor confirmed a significant main effect (F (16, 192) = 32.8, p < 0.00001). No significant effects were confirmed in a factor of regularity (F (1, 12) = 0.24, p = 0.63) and presentation of the MOCR elicitor (F (1, 12) = 0.001, p = 0.978).

**Fig 6:**
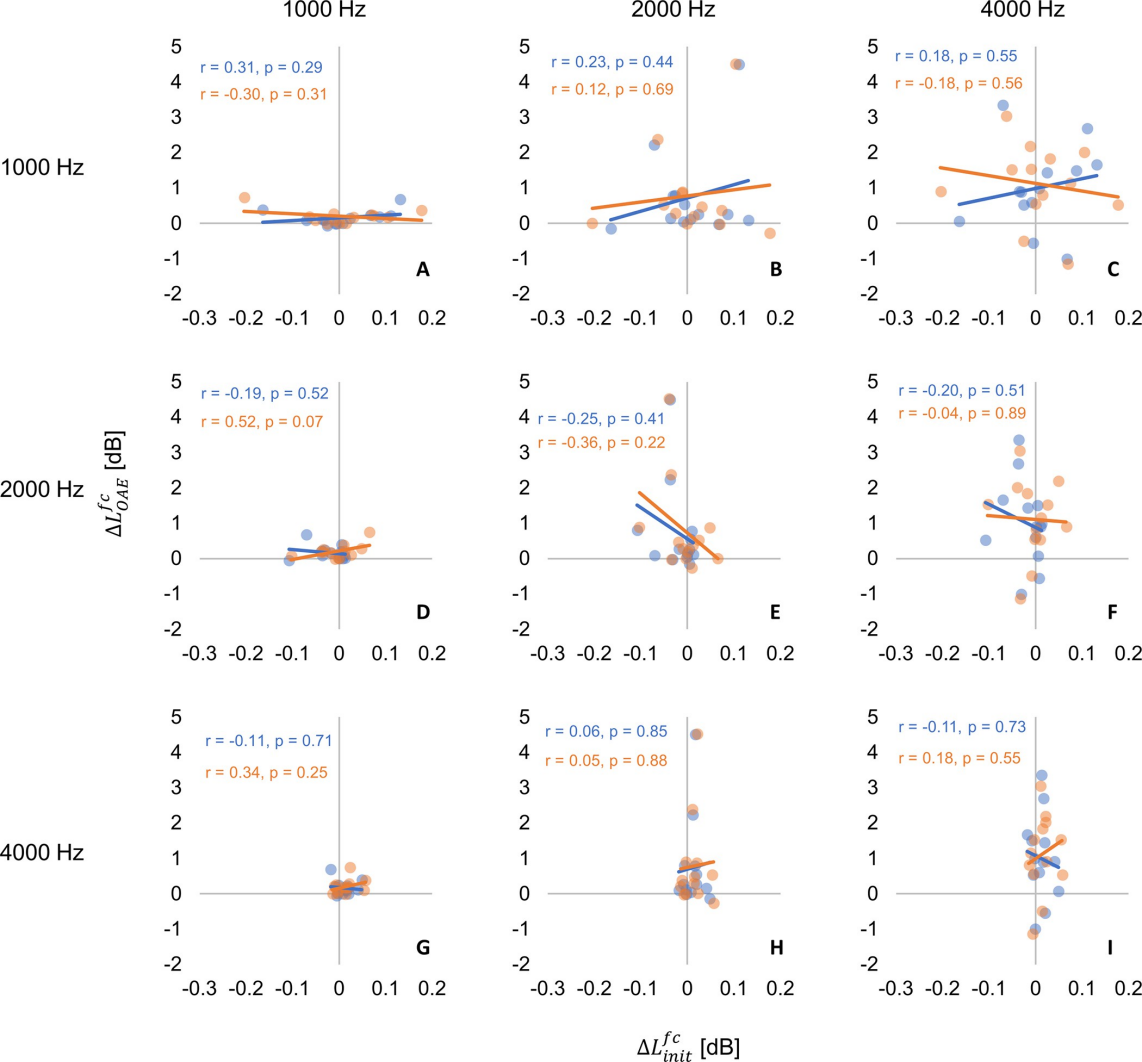
^*fc*^Δ*L*_*OAE*_ vs. ^*fc*^Δ*L*_*init*_ Panels (A–I) separate 1/3-octave bandwidths. In all panels, the x-axis shows the ^*fc*^Δ*L*_*iniit*_ in dB and the y-axis shows the ^*fc*^Δ*L*_*OAE*_ in dB. The frequencies of MEMR and MOCR are shown in panel columns and rows respectively. Comparison frequencies in each panel are the two frequencies intersecting the specific panel. For example, panel (B) compares ^*fc*^Δ*L*_*init*_ [dB] at 1000 Hz and ^*fc*^Δ*L*_*OAE*_ [dB] at 2000 Hz. Significant fits in Predictable and Unpredictable conditions are indicated in blue and orange line respectively. Corresponding Pearson correlation coefficient (r) and the p-value in each regularity conditions are shown as the same color as the fit line at the top of each panel.

Scatter plots of ^*fc*^Δ*L*_*OAE*_ and ^*fc*^Δ*L*_*init*_ for 1000, 2000, 4000 Hz bands are plotted in Fig 6. Only three of the seven frequencies are shown for brevity. Pearson’s correlation coefficients between ^*fc*^Δ*L*_*OAE*_ and ^*fc*^Δ*L*_*init*_ for Predictable and Unpredictable, respectively, were -0.25 ~ 0.31 and -0.36 ~ 0.43, and no correlation between MOCR and MEMR was confirmed for any combination of frequencies.

### Experiment 2

#### Predictable condition

**Fig 7:**
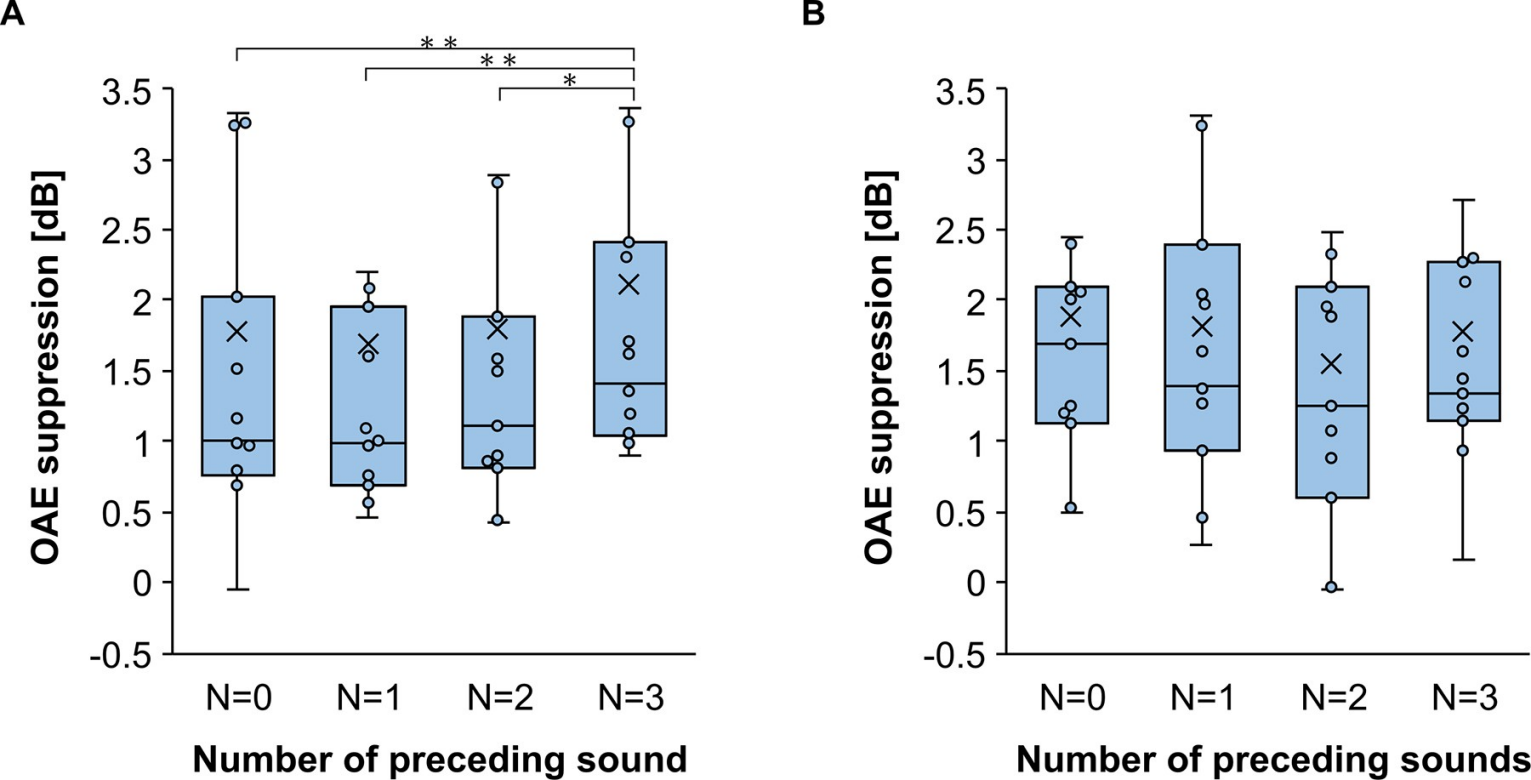
Mean otoacoustic emissions suppression in Experiment 2. (A) Predictable condition. The MOCR induced by the Three-preceding-sounds condition was significantly stronger than that induced by the other conditions. (B) Unpredictable condition. The MOCR did not significantly change with the number of preceding sounds. Error bars represent the standard error of the mean. *p < 0.05, **p < 0.01 (Corrected for multiple comparisons with Benjamini & Hochberg).

Fig 7A shows the decrease in OAE amplitudes caused by the presentation of contralateral noise following the different numbers of preceding tones. The mean reduction in OAE amplitude was 1.79 dB (SD = 1.96) for the Non-preceding-sound condition, 1.69 dB (SD = 1.87) for the One-preceding-sound condition, 1.80 dB (SD = 1.88) for the Two-preceding-sounds, and 2.11 dB (SD = 1.81) for the Three-preceding-sounds condition. A repeated-measures ANOVA with the number of preceding sounds as a factor confirmed a significant main effect (F (3, 30) = 4.83, p = 0.0074). Multiple comparisons using Benjamini & Hochberg method at a 5% level revealed significant differences between the Three-preceding-sounds condition and the other conditions, with t (10) = 2.65, p = 0.048 for the Two-preceding-sounds condition, t (10) = 3.63, p = 0.027 for the One-preceding-sound condition, and t (10) = 2.93, p = 0.045 for the Non-preceding-sound condition. Each participant’s data is shown in S2 Table.

**Fig 8:**
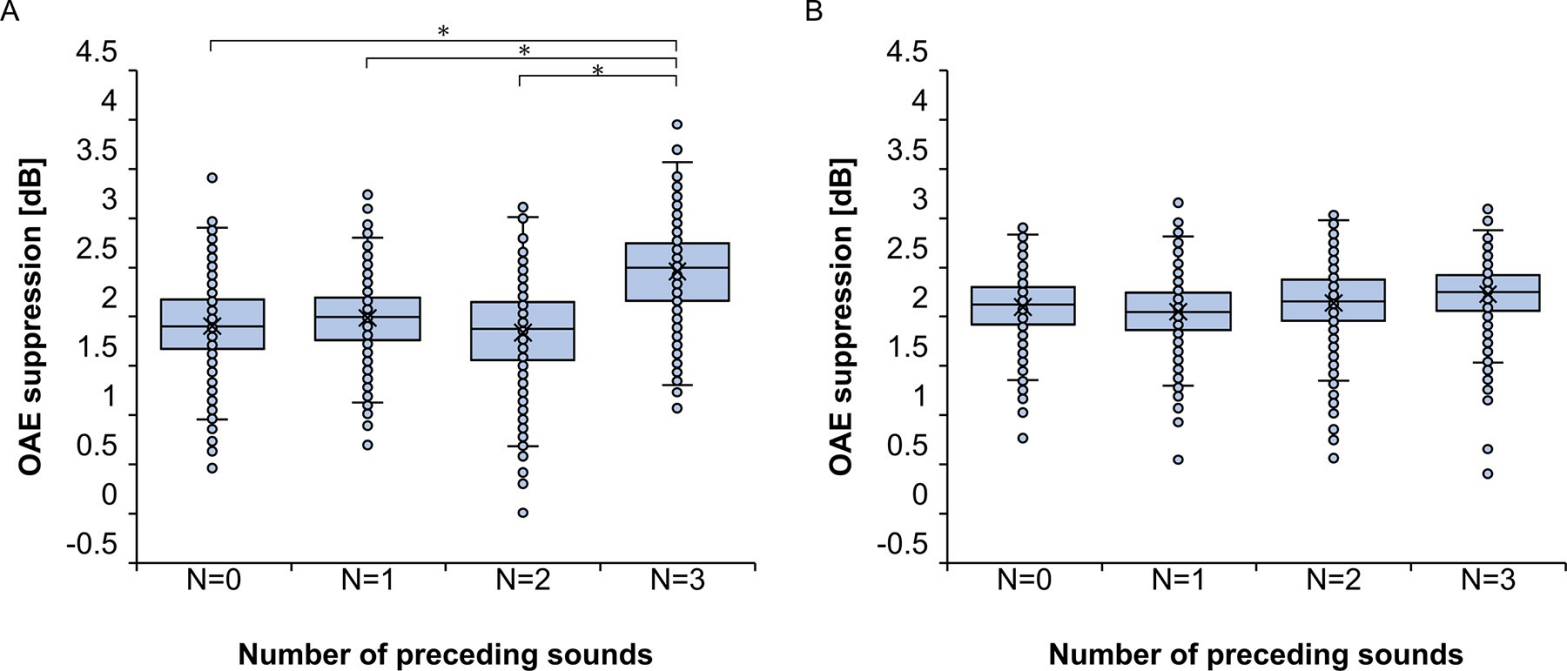
The distributions of the bootstrap means of otoacoustic emission (OAE) suppression in the Experiment 2 for subject 7. (A) Predictable condition. *p<0.05: the lower/upper limit the 95% confidence interval (CI) of the distribution of the difference between bootstrap mean of two conditions was positive/negative. (B) Unpredictable condition.

To evaluate the significance in the difference of the OAE suppression between the Non-, One-, Two- and Three-preceding-sounds conditions within subjects, a bootstrap method was used. Similarly to Experiment 1, we randomly extracted 1200 responses from 1200 responses with repetition and averaged them for the four conditions. This procedure was repeated 1000 time. Fig 8A shows the box-plots of the bootstrap means of OAE suppression for each condition for subject 7. The distribution of the difference between the bootstrap mean of each combination of four conditions were obtained (One-preceding-sounds condition minus Non-preceding-sounds condition, Two- minus Non-preceding-sounds, Three- minus Non-preceding-sounds, Two- minus One-preceding-sounds, Three- minus One-preceding-sounds, Three- minus Two-preceding-sounds). The 95% CI of the distributions was -0.79 ~ 0.97 dB for the One- minus Non-preceding-sounds condition, -1.1 ~ 0.93 dB for the Two- minus One-preceding-sounds condition, 0.00075 ~ 1.97 dB for the Three- minus Two-preceding-sounds condition, -1.16 ~ 0.75 dB for the Two- minus Non-preceding-sounds condition, 0.032 ~ 1.84 dB for the Two- minus One-preceding-sounds condition and 0.045 ~ 2.09 dB for the Three- minus Two-preceding-sounds condition. Comparisons of the lower and upper limit of 95 CI to zero show that the OAE suppression increased at the three-preceding-sounds condition for Subject 7. Similarly, five of the eleven participants showed significantly increased OAE suppression in the three-preceding-sounds condition in the Predictable condition.

To test whether significant MEMR was induced by the MOCR elicitor, the sound pressure of initial portion of the OAE waveforms was compared between with and without contralateral MOCR elicitor. A three-factor repeated-measures ANOVA was performed with the center frequencies, the presentation of the MOCR elicitor and the number of preceding sounds as factors. Although a significant main effect was confirmed in the factor of the center frequency (Predictable: F (16, 160) = 54.8, p < 0.00001), no significant effects were confirmed in a factor of number of preceding sounds (F (3, 3) = 0.45, p = 0.72) and presentation of the MOCR elicitor (F (1, 10) = 2.1, p = 0.18).

**Fig 9:**
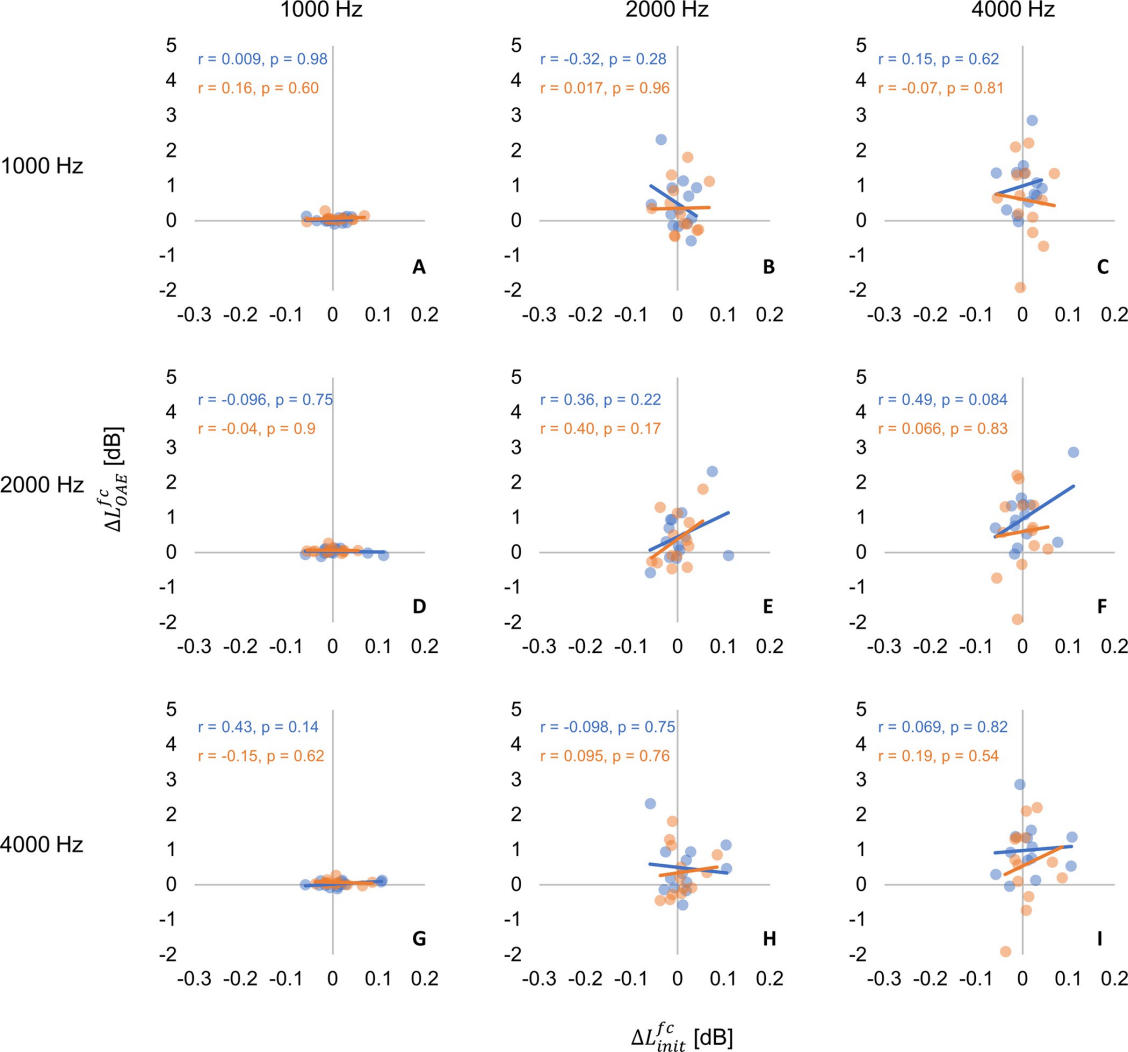
^*fc*^Δ*L*_*OAE*_ vs. ^*fc*^Δ*L*_*init*_ Panels (A–I) separate 1/3-octave bandwidths in Experiment 2. Only three of the seven frequencies in three-preceding-sounds condition are shown. In all panels, the x-axis shows the ^*fc*^Δ*L*_*init*_ in dB and the y-axis shows the ^*fc*^Δ*L*_*OAE*_ in dB. The frequencies of MEMR and MOCR are shown in panel columns and rows respectively. Comparison frequencies in each panel are the two frequencies intersecting the specific panel. For example, panel (B) compares ^*fc*^Δ*L*_*init*_ [dB] at 1000 Hz and ^*fc*^Δ*L*_*OAE*_ [dB] at 2000 Hz. Significant fits in Predictable and Unpredictable conditions are indicated in blue and orange line respectively. Corresponding Pearson correlation coefficient and the p-value in each regularity conditions are shown as the same color as the fit line at the top of each panel.

Fig 9 shows the scatter plots of ^*fc*^Δ*L*_*OAE*_ and ^*fc*^Δ*L*_*init*_ for 1000, 2000, 4000 Hz bands. Only three of the seven frequencies in three-preceding-sounds condition are shown for brevity. No significant correlations were found between ^*fc*^Δ*L*_*OAE*_ and ^*fc*^Δ*L*_*init*_ from 1000 to 4000 Hz at any preceding sound conditions. Pearson’s correlation coefficients between ^*fc*^Δ*L*_*OAE*_ and ^*fc*^Δ*L*_*init*_ for the Non-preceding-sound condition, the One-preceding-sound condition, the Two-preceding-sound condition and the Three-preceding sound condition, respectively, were -0.11 ~ 0.36, -0.017 ~ 0.39, -0.40 ~ 0.42 and -0.32 ~ 0.49 and no significant correlations were found between ^*fc*^Δ*L*_*OAE*_ and ^*fc*^Δ*L*_*init*_ from 1000 to 4000 Hz at any preceding sound conditions.

#### Unpredictable condition

Fig 7B shows the decrease in OAE amplitudes caused by the MOCR elicitor following the different numbers of preceding tone bursts. The mean reduction in OAE amplitude was 1.89 dB (SD = 1.41) for the Non-preceding-sound condition, 1.82 dB (SD = 1.29) for the One-preceding-sound condition, 1.55 dB (SD = 1.32) for the Two-preceding-sound condition, and 1.77 dB (SD = 1.34) for the Three-preceding-sound condition. A repeated-measures ANOVA with the number of preceding sounds as a factor confirmed no significant differences among the conditions (F (3, 30) = 0.88, p = 0.46). Each participant’s data is shown in S2 Table.

The same bootstrap procedure used in the Predictive condition in Experiment 2 was applied to the data in the Unpredictable condition. Fig 8B shows the distribution of the bootstrap mean of the OAE suppression in the Unpredictable conditions for subject 7. The distributions of the difference in the bootstrap mean of the OAE suppression between the four conditions were also calculated. The 95% confidence interval of the distributions was -0.74 ~ 0.67 dB for the One- minus Non-preceding-sounds condition, -0.70 ~ 0.75 dB for the Two- minus Non-preceding-sounds condition, -0.23 ~ 1.08 dB for the Three- minus Non-preceding-sounds condition, -0.72 ~ 0.80 dB for the Two- minus One-preceding-sounds condition, -0.27 ~ 1.08 dB for the Three- minus One-preceding-sounds condition and -0.34 ~ 1.06 dB for the Three- minus Two-preceding-sounds condition. The result showed that there was no significant difference in the OAE suppression among four conditions. All the eleven participants showed same result.

To test whether significant MEMR was induced by the MOCR elicitor, the sound pressure of initial portion of the OAE waveforms was compared between with and without contralateral MOCR elicitor. A three-factor repeated-measures ANOVA was performed with the center frequencies, the presentation of the MOCR elicitor and the number of preceding sounds as factors. Although a significant main effect was confirmed in a factor of the center frequency as a factor confirmed (Predictable: F (16, 160) = 66.1, p < 0.00001). No significant effects were confirmed in a factor of number of preceding sounds (F (3, 3) = 1.51, p = 0.23) and presentation of the MOCR elicitor (F (1, 10) = 2.9, p = 0.12).

Any significant correlations between the ^*fc*^Δ*L*_*OAE*_ and ^*fc*^Δ*L*_*init*_ was found at all the center frequency. Pearson’s correlation coefficients between ^*fc*^Δ*L*_*OAE*_ and ^*fc*^Δ*L*_*init*_ for the Non-preceding-sound condition, the One-preceding-sound condition, the Two-preceding-sound condition and the Three-preceding sound condition, respectively, were -0.44 ~ 0.48, -0.26 ~ 0.35, -0.25 ~ 0.27 and -0.15 ~ 0.19 and no significant correlations were found between ^*fc*^Δ*L*_*OAE*_ and ^*fc*^Δ*L*_*init*_ from 1000 to 4000 Hz at any preceding sound conditions.

## Discussion

In this study, we reported the effects of rhythmic temporal expectations on the MOCR. The MOCR strength was in the range of 0–4 dB which is consistent with previous studies [13, 40]. Experiment 1 showed that the MOCR was stronger when the timing of the MOCR elicitor was predictable than when it was unpredictable, based on the regularity of the preceding sound sequence. The preceding tone bursts were presented to modulate the predictability of the timing of the MOCR elicitor occurrence. However, the preceding tone bursts themselves could also evoke MOCR. Although the effect is thought to be attenuated before the MOCR elicitor occurrence, given the MOCR effects decayed exponentially with a time constant of approximately 160 ms [41], their faint residuals may influence the response to the MOCR elicitor. To avoid the unbalanced effects of the residual between the conditions, in the Unpredictable condition, we extracted the blocks whose ISI between the last tone burst and the MOCR elicitor was equal to that in the Predictable condition. However, it is still possible that the difference of the MOCR strength between the two conditions could be accounted for by difference in temporal energy integration driven by the first and second tone burst. As MOCR increases exponentially at stimulus onset with a time constant of around 100 ms and decreases exponentially at stimulus offset with a time constant of from 300 ms [41], which is in line with the concept of temporal energy integration [18]. This temporal energy integration would underlie the phenomenon that modulated noise elicits significantly smaller MOCR relative to unmodulated noise despite the equal RMS [42, 43]. In the case of the present study, tone bursts presented with smaller average ISI may drive more temporal energy integration, i.e., stronger MOCR, than those presented with larger average ISI. However, in this experiment, to avoid the unbalanced effect of temporal energy integration, the number of 250, 500 and 1000-ms ISI was counterbalanced within one measurement secession. These procedures assure that the stronger MOCR in the predictable condition cannot be explained by the effect of the MOCR induced by the preceding tone bursts.

In Experiment 2, we examined the effects of the number of preceding sound sequences on the regularity-based MOCR enhancement. In the Predictable condition, the MOCR was significantly stronger during the three-preceding sounds condition compared with the single- and two-preceding sounds conditions. Namely, at least three preceding sounds were required to drive the regularity-based enhancement of the MOCR. This also suggests that the MOCR was not automatically enhanced by a single stimulus presented immediately before the MOCR elicitor, but rather that the MOCR was enhanced by the regularity of the preceding sound sequence.

The dependencies on the number of preceding sounds may reflect the hazard rate of the MOCR elicitor occurrence. The measurement sessions did not include any trials without MOCR elicitor presentation. Meaning, the MOCR elicitor certainly appeared after the occurrence of three preceding tone bursts. Hence the conditional probability of the MOCR elicitor occurring at a given time, namely hazard rate, should increase as the number of preceding tone bursts occur up to that time and reach the maximum after the third preceding tone burst occurs, given that the MOCR elicitor had not yet occurred. The increased hazard rate might enhance the MOCR in the Three-preceding-sounds condition compared to the other condition. However, in the Unpredictable condition, the MOCR did not change with the number of preceding sounds, which suggests that the MOCR enhancement in the three-preceding sounds condition in the Predictable condition cannot be explained by hazard rate effects.

The findings from experiment 2 suggests that three tone bursts drive more accurate prediction than single or two tone bursts. It would be possible that more than three bursts elicit stronger MOCR because an increase of the number of preceding tone bursts may improve the accuracy of the prediction on the timing of the MOCR elicitor arrival. Mansuri et al. (2022) reported that accuracy of rhythm perception was higher as the number of beats increased [44]. Considering that Mansuri et al. (2022) varied the number of beats form 3 to 8, it is reasonable to suppose that more preceding tone bursts would drive larger enhancement of MOCR, although the number of preceding tone bursts in the present study was limited to less than three. Therefore, it is possible that the MOCR strength saturates under conditions with more than about three preceding tones, but further study is needed in this regard.

We evaluated the MOCR strength by OAE suppression induced by MOCR elicitor presentation. One may think that the changes in OAE suppression associated with temporal regularity can be attributed to MEMR changes. Thus, we determined the effects of MEMR by evaluating the level differences of the initial part of the OAE waveforms before and after the MOCR elicitor (^*fc*^Δ*L*_*init*_). The initial portions of the waveforms are dominated by the ringing of the click stimulus inside the ear canal. Therefore, the changes associated with the MOCR elicitor presentation can be assumed to reflect MEMR-related changes in the reflectance of the ear drum, or middle ear transmission. However, we did not find any significant differences in ^*fc*^Δ*L*_*init*_ among the conditions at any center frequency of 1/3-octave bands and any significant correlation between MOCR strength and MEMR strength, which supports the notion that changes in OAE suppression among conditions reflect the changes in MOCR-associated with temporal predictability. On the other hand, the bootstrap analysis reveals that the inter-trial variability of MOCR strength was large and approximately only 50% of participants exhibited a significant MOCR enhancement associated with temporal predictability. This result suggests that the effect of temporal predictability is weak and more statistical power with large number of trials and participants is required to capture the within-subject MOCR changes associated with temporal predictability.

Few studies have investigated the effects of prediction on peripheral auditory processing. Otsuka et al. reported that the MOCR was enhanced by presenting a visual cue task conveying the timing of the target appearance immediately before the stimulus [13]. The result suggests that endogenous temporal orientation influences peripheral auditory processing. It was also reported that OAE amplitude increased when the frequency of a behaviorally relevant upcoming sound was predicted from patterns of frequency changes in isochronous tone sequences [30]. However, this study is the first to show evidence that the descending pathway conveys a rhythmic, presumably exogenous, temporal expectation of an upcoming sound to the first stage of auditory processing.

In cortical regions, it has been reported that the regularity of sound sequences improves the entrainment of δ-band oscillations to the stimuli [9–11]. Dragicevic, et al. [27] showed significant correlations between the fluctuations of OAE levels and slow rate EEG oscillations (1–7 Hz) during cognitive tasks, which extends the role of the oscillatory activity network during cognition in neural systems to the receptor level. The oscillatory modulation of peripheral activities could be achieved by the corticofugal pathways which originate in the cortical regions and project to the MOC bundle via subcortical nuclei [15, 20, 21]. The corticofugal pathways can also bridge the cortical predictive processing, revealed as enhanced cortical entrainments, and MOCR enhancement associated with rhythmic temporal expectation, which was observed in our experiments.

Temporal predictability has been reported to suppress cortical potentials [45, 46] and to enhance repetition suppression, which is a possible human electrophysiological counterpart of stimulus-specific adaptation (SSA) [47], which a plausible neural substrate for predictive encoding [48–51]. This is in line with the predictive coding hypothesis, in which the neural responses to expected stimuli should be reduced [52]. Anderson and Malmierca (2013) further showed that inactivation of the auditory cortex modulates SSA of cells in subcortical regions [53]. The descending pathway may be related to forming or facilitating predictive processing at subcortical levels [51]. Enhanced MOCR associated with temporal predictability, which leads to increased suppression of the cochlear response, can also be understood as a part of a prediction-based corticofugal inhibition network underling the predictive coding framework.

Concerning optimization, the MOCR enhancement based on temporal regularity is reasonable. The MOCR inhibits OHC motility and improves the detection of signals in noisy environments by preventing the adaption of auditory nerves to the noise and maintaining their responsiveness to upcoming targets, known as the antimasking effect [16, 17, 54]. The suppression induced by the MOCR protects the sensory system from acoustic overexposure [19, 55]. Stronger suppression facilitates noise protection and antimasking effects but disturbs the detection of faint signals. In this sense, the time-specific exertion of the MOCR enhancement at a relevant time point could be a reasonable solution to the dilemma.

## Supporting information

S1 Table Each participant’s data of experiment 1. (XLSX)

S2 Table Each participant’s data of experiment 2. (XLSX)
